# Public views on ethical issues in healthcare artificial intelligence: protocol for a scoping review

**DOI:** 10.1186/s13643-022-02012-4

**Published:** 2022-07-15

**Authors:** Emma Kellie Frost, Rebecca Bosward, Yves Saint James Aquino, Annette Braunack-Mayer, Stacy M. Carter

**Affiliations:** Australian Centre for Health Engagement, Evidence and Values, School of Health and Society, Faculty of the Arts, Social Sciences, and Humanities, University of Wollongong, Northfields Ave, Wollongong, NSW 2522 Australia

**Keywords:** Artificial Intelligence, AI ethics, Healthcare AI, Patients, Publics

## Abstract

**Background:**

In recent years, innovations in artificial intelligence (AI) have led to the development of new healthcare AI (HCAI) technologies. Whilst some of these technologies show promise for improving the patient experience, ethicists have warned that AI can introduce and exacerbate harms and wrongs in healthcare. It is important that HCAI reflects the values that are important to people. However, involving patients and publics in research about AI ethics remains challenging due to relatively limited awareness of HCAI technologies. This scoping review aims to map how the existing literature on publics’ views on HCAI addresses key issues in AI ethics and governance.

**Methods:**

We developed a search query to conduct a comprehensive search of PubMed, Scopus, Web of Science, CINAHL, and Academic Search Complete from January 2010 onwards. We will include primary research studies which document publics’ or patients’ views on machine learning HCAI technologies. A coding framework has been designed and will be used capture qualitative and quantitative data from the articles. Two reviewers will code a proportion of the included articles and any discrepancies will be discussed amongst the team, with changes made to the coding framework accordingly. Final results will be reported quantitatively and qualitatively, examining how each AI ethics issue has been addressed by the included studies.

**Discussion:**

Consulting publics and patients about the ethics of HCAI technologies and innovations can offer important insights to those seeking to implement HCAI ethically and legitimately. This review will explore how ethical issues are addressed in literature examining publics’ and patients’ views on HCAI, with the aim of determining the extent to which publics’ views on HCAI ethics have been addressed in existing research. This has the potential to support the development of implementation processes and regulation for HCAI that incorporates publics’ values and perspectives.

**Supplementary Information:**

The online version contains supplementary material available at 10.1186/s13643-022-02012-4.

## Background

Recent years have seen the development and introduction of a number of artificial intelligence (AI) enabled technologies for healthcare. AI is a term which encompasses diverse computational technologies, making it challenging to define: prominent definitions include that AI is ‘a collection of interrelated technologies used to solve problems that would otherwise require human cognition’ [1], or that AIs are technologies with the ability to ‘perform tasks to achieve defined objectives without explicit guidance from a human being’ [2]. Broad in application, AI technologies arrive with optimistic promises of transforming the patient experience. Many of these modern developments are the result of innovations in machine learning (ML), a branch of AI focussed on developing algorithms which learn from examples [3]. So far, advocates of healthcare AI (HCAI) have promised the technology will improve the accuracy of screening and diagnosis [4], increase the availability of care in remote regions [5], and free up physicians’ time so that they can engage more with patients [6].

Alongside innovations in HCAI, there is also a growing field of AI ethics which cautions against uncritical implementation of HCAI and raises questions about its regulation [7]. ML technologies pose new risks and challenges to healthcare: some ML algorithms have been shown to produce biased outcomes [8], many ML technologies are ‘black boxes’ where the reasons behind an algorithm’s output cannot be interpreted [9], and questions remain about how existing liability structures in medicine will effectively manage errors made by deployed ML technologies [10]. AI development also continues to be dominated by large private companies that have been criticised for failing to engage in meaningful conversations about the ethics of their products and research [11].

Publics may be both beneficiaries of new HCAI technologies and the greatest sufferers of AI-related harms [12]. Patients and publics are important voices in developing effective and ethical AI governance, but engaging patients and publics meaningfully in research about ethical HCAI is challenging. Most people have no firsthand experience with HCAI, and some are unfamiliar with the concept of AI in general [13]. Publics may have limited understanding of how HCAI may be implemented, and limited knowledge about the potential wrongs and harms that could arise from implementing HCAI. To ensure that HCAI has a positive impact on patients, it is crucial that AI ethics reflects the values that are important to people [12, 14], but it remains unclear how this should be achieved.

The aim of this review is to determine how common and emerging themes in HCAI ethics are addressed by the existing literature on publics’ and patients’ views on machine learning in healthcare.

## Methods/design

Scoping reviews are an effective method for exploring the range and extent of literature on a given topic [15]. Our work will follow the framework proposed by Arksey and O’Malley [16] with modifications from Levac and colleagues [15]. Their six recommended steps include (a) identifying the research question; (b) identifying relevant studies; (c) study selection; (d) charting the data; (e) collating, summarising, and reporting the results; and (f) consultation. The following sections will address each of these steps in greater detail. In preparing this protocol, we completed a PRISMA-P checklist to ensure all necessary details have been reported (Additional file 1).

### Stage 1: Identifying the research questions

Our review will address the question, *to what extent, and how, are HCAI ethics issues addressed in the existing literature on publics’ views on machine learning applications of HCAI*. Our objectives are (1) to explore whether and how research on public views regarding HCAI has included investigation of public views on HCAI ethics (2) to describe study participants’ perspectives on HCAI ethics issues.

### Stage 2: Identifying relevant literature

We developed a search query using the Population-Intervention-Context-Outcome (PICO) format. An initial search on Google Scholar helped to identify similar terms which were used to develop a comprehensive search query for published literature (Table 1).

**Table 1:** Grid of terms describing search strategy

Population	(“women”[MeSH] OR”men”[MeSH] OR”patients”[MeSH] OR”public”[tiab] OR”publics”[tiab] OR”consumers”[tiab] OR”population”[tiab] OR”participants”[tiab] OR”consumer”[tiab] OR”participant”[tiab] OR”patient”[tiab] OR”women”[tiab] OR”men”[tiab] OR”patients”[tiab]) AND
Intervention	(“artificial intelligence”[MeSH] OR”machine learning”[MeSH] OR”artificial intelligence”[tiab] OR”machine learning”[tiab] OR”deep learning”[tiab] OR”neural network”[tiab] OR”neural networks”[tiab]) AND
Context	(“delivery of health care”[MeSH] OR”health services”[MeSH] OR”mass screening”[MeSH] OR”diagnosis”[MeSH] OR”therapeutics”[MeSH] OR”screening”[tiab] OR”clinical”[tiab] OR”healthcare”[tiab] OR”health care”[tiab] OR”surgery”[tiab] OR”diagnostics”[tiab] OR”diagnostic”[tiab] OR”diagnosis”[tiab] OR”health services”[tiab] OR”therapeutics”[tiab]) AND
Outcome	(“attitude”[MeSH] OR”perception”[MeSH] OR”perspective”[tiab] OR”perspectives”[tiab] OR”preference”[tiab] OR”preferences”[tiab] OR”priorities”[tiab] OR”intention”[tiab] OR”intentions”[tiab] OR”attitude”[tiab] OR”perception”[tiab])

We will use a systematic search strategy to find relevant articles for inclusion in the study. Databases to be searched are PubMed, Scopus, Web of Science, CINAHL, and Academic Search Complete. To find relevant grey literature, we will screen the first ten pages of a Google Advanced search. We will examine the reference lists of included studies to find any publications that were missed in the initial searches. All studies collected through the search project will be imported into a Zotero library.

### Stage 3: Study selection

After the search is completed, all studies will be screened for eligibility. EF will be responsible for conducting the search and managing the data. First, duplicates will be removed using the deduplication module from the Systematic Review Assistant [17], and then the remaining files will be exported to MS Excel for the screening process. MS Excel will allow reviewers to easily indicate a study’s inclusion or exclusion, as well as keep notes about any uncertainties for discussion.

The first stage of screening will exclude irrelevant articles based on their title and abstract. Screening will be conducted based on a set of criteria defined below. The first reviewer (EF) will screen the first 10% of articles, including all articles that are potentially relevant based on their title and abstract and excluding articles that are clearly irrelevant. Of this 10%, EF will construct a sample of approximately 40 articles marked for inclusion, and 60 articles marked for exclusion. A second reviewer (RB) will screen this sample of 100 articles and compare results with EF. Results will be discussed with the team and inclusion criteria will be modified if necessary. Once any issues have been resolved, EF will conduct the initial screening on the remainder of the studies.

After initial screening is completed, excluded articles will be removed and full article texts will be collected for the remaining studies. The two-reviewer screening process will be repeated on a random sample of 10% of the full texts. Differences will be discussed and resolved, and modifications will be made to the inclusion criteria accordingly. Inter-rater scores will be generated to quantify agreeance between reviewers. Once the inclusion criteria are finalised, EF will conduct the remainder of the full-text screening.

### Inclusion criteria

Articles will be screened against a set of inclusion criteria developed by the team. These criteria may be modified throughout the screening process. Initial design of the criteria was guided by the JBI guidelines for scoping reviews [18]. This guide states that inclusion criteria should address (i) the types of participants, (ii) concept, (iii) context, and (iv) types of evidence sources.

#### Types of participants

Studies will be included if research participants are recruited as publics, patients (or their unpaid/familial carers), or healthcare consumers. If studies recruit professionals (e.g. physicians, nurses, policymakers, or professional carers) along with publics, they will be included so long as the data related to patients/publics can be extracted.

#### Concept

Studies must address publics’ or patients’ views on HCAI. In this case, we utilise the term “views” to refer to the various ways participants contribute to social research. Included studies may, for example, quantitatively measure participants’ attitudes toward HCAI, or qualitatively examine participants’ perspectives on HCAI (or an application thereof). Studies will be excluded if the participants’ contribution to the research does not involve sharing views (e.g. studies only measuring whether a particular HCAI tool has improved participant outcomes in a certain area).

Studies will be included if the research addresses machine learning in patient- or (general) public-facing health care or services. An included study may address machine learning in healthcare or services in a specific field (e.g. patients’ perspectives on AI for breast screening, or publics’ attitudes toward AI-enabled mobile phone apps for skin cancer detection).

Studies will be excluded if they only address AI technologies that are not within the machine learning branch of AI. For example, studies only examining participants’ views on care robots or expert systems would be excluded. Studies will be excluded if they only address AI in non-patient/public-facing health applications. For example, studies addressing AI used only to manage bills and claims processing in hospitals would be excluded. Studies will be excluded if they only address non-health applications of AI.

#### Context

Studies from any geographical location will be included, so long as the manuscript can be assessed in English. Only studies published between 1 January 2010 and 15 September 2021 will be included. This time period has seen the introduction of modern approaches such as deep learning, convolutional neural networks, and natural language processing into HCAI research [6]. These new approaches are the source of much of the current interest and investment in HCAI, and introduce a number of new potential challenges and harms [7].

#### Types of evidence sources

Only primary research studies will be included in this review. Studies will not be excluded based on method. There will be no restrictions on study design.

Studies will be excluded if they are only available in a language other than English, if they do not address AI in a patient-facing healthcare context or if the study participant profile does not include patients or publics.

### Stage 4: Charting the data

We have designed a coding framework to capture information on whether and how studies address a series of AI ethics concerns.^1^ Whilst a number of different frameworks were reviewed [19, 20], the coding framework is primarily based on Fjeld and colleagues’ [21] analysis of a series of AI ethics guidelines. Fjeld et al. identified seven domains that were frequently addressed in AI ethics frameworks: (1) *privacy*, (2) *accountability*, (3) *safety and security*, (4) *transparency and explainability*, (5) *fairness and non-discrimination*, (6) *human control over technology*, and (7) *professional responsibility*. To capture more detailed data on where each of these ethical issues were addressed, we separated the concepts of ‘*safety’* and ‘*security’*, and ‘*transparency’* and ‘*explainability’* into individual code categories (Table 2).

**Table 2:** Adaptation of AI ethics frameworks for data extraction

Concept	Reference(s)	Description
Privacy	[21]	Whether study addresses publics’ views on privacy, consent, control over the use of data, and/or right to erasure
Accountability	[21]	Whether study addresses publics’ views on legal liability and responsibility for rectification when algorithms perform poorly
Safety	[21]	Whether study addresses publics’ views on the consistency and accuracy of algorithms’ performance, or the perceived safety of using AI in healthcare and services
Security	[21]	Whether study addresses publics’ views on algorithms’ vulnerability to nefarious third parties
Transparency	[21]	Whether study addresses publics’ views on the transparency of AI development and implementation, and/or the importance of disclosing that AI is being used
Explainability	[21]	Whether study addresses publics’ views on algorithmic explainability, black box algorithms, and/or the importance of patients’ and physicians’ ability to understand the reasons behind an algorithm’s decision
Fairness and non-discrimination	[21]	Whether study addresses publics’ views on algorithmic bias, fairness in algorithmic decision-making, and/or inclusivity in AI design
Human control over technology	[21]	Whether study addresses publics’ perspectives on the extent to which humans should review automated decisions, and whether people should be able to opt out of algorithm-informed decisions
Professional responsibility	[21]	Whether study addresses publics’ perspectives on professionals’ roles in ensuring that algorithms are accurate, perform well, and do not cause harms
Power	[14, 22]	Whether study addresses publics’ perspectives on the impact of AI on existing power structures in society. E.g. concerns about AI reinforcing existing power structures, the inclusivity of AI governance and regulation, and/or the development of AI technologies which primarily benefit the Global North
Environmental wellbeing	[23]	Whether study addresses publics’ views on the environmental impacts of AI, including e-waste, energy consumption, and materials
Societal wellbeing	[23]	Whether study addresses publics’ views on whether algorithms are being created and implemented for broader social good
Ethical governance	[24]	Whether study addresses publics’ views on the suitability of existing legal structures, or the need for new structures, to manage the ethical issues associated with HCAI

We added four additional concepts to the framework. The first, *power*, has become a more common point of discussion in AI ethics frameworks recently, to assess how AI development and governance structures are reinforcing existing power dynamics and failing to redistribute power to marginalised groups [14, 22]. The second, *environmental wellbeing*, addresses the environmental impacts of AI development including energy usage, materials, and e-waste [22, 23]. *Societal wellbeing* addresses whether technological development is being implemented for social good [23]. Finally, *ethical governance* addresses whether existing governance structures are suitable to manage the ethical issues associated with HCAI.

Additional information about study design and methods will also be collected, including detailed notes on study design. We will use MS Excel to chart the data from the studies. The initial data extraction tool (Additional file 2) covers the key areas recommended by Arksey and O’Malley [16], the ethics framework, and the additional information about study design. In adhering to recommendations from Levac et al. [15], we will modify this tool progressively throughout the data collection process. Initially, a random 10% of the included studies will be selected and coded by two coders (EF & RB) and any differences will be resolved in consultation with the research team. We will make changes to the data extraction tool if necessary. The remainder of the charting will be conducted by EF.

### Stage 5: Collating, summarising, and reporting the results

We will collate results into tabular format for analysis. Guided by Arksey and O’Malley’s [16] recommendations, analysis will begin with descriptive quantitative reporting where it is appropriate (e.g. the number of studies which address each HCAI ethics issue in the framework).

Our reporting will synthesise publics’ and patients’ views on each of the HCAI ethics issues in Table 2. Given the inclusion criteria for this review, we are likely to collect studies with diverse designs. In some cases, direct quantitative comparison between studies may be possible. In other cases, studies with different methodological designs may be compared with one another. Where studies do not allow for direct comparisons, our results will report narrative descriptions and comparisons, noting how a study’s framing, aims, and contexts might influence the information collected. This synthesis methodology will be refined based on the types of studies collected.

## Discussion

This review may have some limitations. Firstly, scoping reviews are designed to map the literature in a topic and are not designed to assess the quality of included studies [15]. The quality of the studies included in this review will not be systematically assessed. Secondly, it is possible that relevant studies will not be captured by the search strategy defined in this protocol. We will conduct a systematic pearling process on relevant identified studies to ensure as many relevant articles are identified as possible. Finally, findings will be limited to studies published in English, which may exclude relevant articles published in other languages. We will reflect on the impact of these limitations, as well as discuss any other arising limitations, in the reporting of our results.

The more widespread use of HCAI technologies is often described as inevitable [6]. However, implementation of HCAI may exacerbate certain harms in healthcare [7]. Although patients and publics are likely to be the greatest sufferers of HCAI-related harms, involving patients and publics in meaningful research about AI ethics remains challenging [13].

To date, the extent to which patients and publics are involved in research about HCAI ethics is unclear. This review will examine where existing research has involved patients and publics in research about a series of HCAI ethics issues. In doing so, we will describe patients’ and publics’ views on each HCAI ethics issue, and highlight potential gaps, or areas of HCAI ethics where research with patients and publics is limited. The results from this review will be important to understanding where further effort is required to involve patients and publics in research about HCAI ethics. Such an effort is crucial to ensuring that HCAI is implemented safely and effectively.

## Supplementary Information

**Additional file 1.**

**Additional file 2.**

## Data Availability

No datasets were generated or used for this paper.

## Abbreviations

AI: Artificial intelligence
HCAI: Healthcare artificial intelligence
PICO: Population-Intervention-Context-Outcome

## Footnotes

1 AI ethics considerations inevitably overlap with legal considerations, as ethics and the law are both normative domains which share some common concerns. We do not make a strong demarcation between ethical and legal considerations in this protocol, but we do approach normative concepts from the perspective of ethics rather than the law.

## References

[1] Walsh T, Levy N, Bell G, Elliott A, Maclaurin J, Mareels I, et al. The effective and ethical development of artificial intelligence: an opportunity to improve our wellbeing. The Australian Council of Learned Academies; 2019.

[2] Hajkowicz S, Karimi S, Wark T, Chen C, Evans M, Rens N, et al. Artificial Intelligence: solving problems, growing the economy and improving our quality of life. CSIRO, Data61; 2019 p. 68.

[3] Jordan MI, Mitchell TM (2015). Machine learning: trends, perspectives, and prospects. Science.

[4] Esteva A, Topol E (2019). Can skin cancer diagnosis be transformed by AI?. Lancet.

[5] Sechopoulos I, Mann RM (2020). Stand-alone artificial intelligence - the future of breast cancer screening?. Breast.

[6] Topol E (2019). Deep Medicine: How artificial intelligence can make healthcare human again.

[7] Carter SM, Rogers W, Win KT, Frazer H, Richards B, Houssami N (2020). The ethical, legal and social implications of using artificial intelligence systems in breast cancer care. The Breast.

[8] Obermeyer Z, Powers B, Vogeli C, Mullainathan S (2019). Dissecting racial bias in an algorithm used to manage the health of populations. Science.

[9] Dilsizian ME, Siegel EL (2018). Machine meets biology: a primer on artificial intelligence in cardiology and cardiac imaging. Curr Cardiol Rep.

[10] Grote T, Berens P (2020). On the ethics of algorithmic decision-making in healthcare. J Med Ethics.

[11] Holzmeyer C (2021). Beyond ‘AI for Social Good’ (AI4SG): social transformations—not tech-fixes—for health equity. Interdisc Sci Rev.

[12] Laï MC, Brian M, Mamzer MF (2020). Perceptions of artificial intelligence in healthcare: findings from a qualitative survey study among actors in France. J Transl Med.

[13] Young AT, Amara D, Bhattacharya A, Wei ML (2021). Patient and general public attitudes towards clinical artificial intelligence: a mixed methods systematic review. The Lancet Digital Health.

[14] Hickok M (2021). Lessons learned from AI ethics principles for future actions. AI Ethics.

[15] Levac D, Colquhoun H, O’Brien KK (2010). Scoping studies: advancing the methodology. Implementation Sci.

[16] Arksey H, O’Malley L (2005). Scoping studies: towards a methodological framework. Int J Soc Res Methodol.

[17] Rathbone J, Carter M, Hoffmann T, Glasziou P (2015). Better duplicate detection for systematic reviewers: evaluation of Systematic Review Assistant-Deduplication Module. Syst Rev.

[18] Peters MDJ, Godfrey CM, Khalil H, McInerney P, Parker D, Soares CB (2015). Guidance for conducting systematic scoping reviews. JBI Evidence Implementation.

[19] Floridi L, Cowls J. A unified framework of five principles for AI in society. Harvard Data Science Review. 2021 [cited 2022 Jun 16]; Available from: https://hdsr.mitpress.mit.edu/pub/l0jsh9d1

[20] Jobin A, Ienca M, Vayena E. Artificial intelligence: the global landscape of ethics guidelines. 2019;42.

[21] Fjeld J, Achten N, Hilligoss H, Nagy A, Srikumar M. Principled artificial intelligence: mapping consensus in ethical and rights-based approaches to principles for AI. SSRN J. 2020 [cited 2021 Sep 2]; Available from: https://www.ssrn.com/abstract=3518482

[22] Crawford K. Atlas of AI: power, politics, and the planetary costs of artificial intelligence. Atlas of AI. Yale University Press; 2021 [cited 2021 Sep 15]. Available from: https://www.degruyter.com/document/doi/10.12987/9780300252392/html

[23] European Commission. Ethics guidelines for trustworthy AI | Shaping Europe’s digital future. 2019 [cited 2021 Sep 2]. Available from: https://digital-strategy.ec.europa.eu/en/library/ethics-guidelines-trustworthy-ai

[24] Guan J (2019). Artificial intelligence in healthcare and medicine: promises, ethical challenges and governance. Chin Med Sci J.

