# Supplementary material for: Public views on ethical issues in healthcare artificial intelligence: protocol for a scoping review
Source: Syst Rev. 2022 Jul 15;11:142. doi: 10.1186/s13643-022-02012-4 (PMC9288036; doi:10.1186/s13643-022-02012-4)
Supplement: Supplementary file 2 — Additional file 2. [file 13643_2022_2012_MOESM2_ESM.docx]

# Additional file 2 – Full data extraction tool

| Variable | Description |
| --- | --- |
| Title | Title of the publication |
| Surname | Surname of first author |
| Year | Year of publication |
| Qual/Quant | Whether the publication documents qualitative, quantitative, or mixed methods research. Additional 'other' category for research which falls outside of these categories |
| Study_design | Description of method |
| Country | Country (or countries) in which research was conducted |
| Objectives | Stated objectives of the research |
| Sample | Final sample size |
| Population | Description of the inclusion criteria for participation (e.g. women with lived experience of breast cancer) |
| Healthcare_setting | Description of the types of HCAI addressed by the study (e.g. machine learning in psychiatry) |
| AI_ethics_questions | Copy or description of all survey/interview questions which sought participants' opinions on AI ethics |
| Privacy_design | Notes on how the research was designed to elicit opinions on privacy. Record N/A if study design does not address privacy |
| Privacy_participants | Notes on the answers expressed by participants about privacy. Record N/A if participants do not express views on privacy. |
| accountability_design | Notes on how the research was designed to elicit opinions on accountability. Record N/A if study design does not address accountability |
| accountability_participants | Notes on the answers expressed by participants about accountability. Record N/A if participants do not express views on accountability. |
| safety_design | Notes on how the research was designed to elicit opinions on safety. Record N/A if study design does not address safety |
| safety_participants | Notes on the answers expressed by participants about safety. Record N/A if participants do not express views on safety. |
| security_design | Notes on how the research was designed to elicit opinions on security. Record N/A if study design does not address security |
| security_participants | Notes on the answers expressed by participants about security. Record N/A if participants do not express views on security. |
| transparency_design | Notes on how the research was designed to elicit opinions on transparency. Record N/A if study design does not address transparency |
| transparency_participants | Notes on the answers expressed by participants about transparency. Record N/A if participants do not express views on transparency. |
| explainability_design | Notes on how the research was designed to elicit opinions on explainability. Record N/A if study design does not address explainability |
| explainability_participants | Notes on the answers expressed by participants about explainability. Record N/A if participants do not express views on explainability. |
| fairness_and_non-discrimination_design | Notes on how the research was designed to elicit opinions on fairness and non-discrimination. Record N/A if study design does not address fairness and non-discrimination |
| fairness_and_non-discrimination_participants | Notes on the answers expressed by participants about fairness and non-discrimination. Record N/A if participants do not express views on fairness and non-discrimination. |
| human_control_over_technology_design | Notes on how the research was designed to elicit opinions on human control over technology. Record N/A if study design does not address human control over technology |
| human_control_over_technology_participants | Notes on the answers expressed by participants about human control over technology. Record N/A if participants do not express views on human control over technology. |
| professional_responsibility_design | Notes on how the research was designed to elicit opinions on professional responsibility. Record N/A if study design does not address professional responsibility |
| professional_responsibility_participants | Notes on the answers expressed by participants about professional responsibility. Record N/A if participants do not express views on professional responsibility. |
| power_design | Notes on how the research was designed to elicit opinions on power. Record N/A if study design does not address power |
| power_participants | Notes on the answers expressed by participants about power. Record N/A if participants do not express views on power. |
| environmental_wellbeing_design | Notes on how the research was designed to elicit opinions on environmental wellbeing. Record N/A if study design does not address environmental wellbeing |
| environmental_wellbeing_participants | Notes on the answers expressed by participants about environmental wellbeing. Record N/A if participants do not express views on environmental wellbeing. |
| societal_wellbeing_design | Notes on how the research was designed to elicit opinions on societal wellbeing. Record N/A if study design does not address societal wellbeing |
| societal_wellbeing_participants | Notes on the answers expressed by participants about societal wellbeing. Record N/A if participants do not express views on societal wellbeing. |
